# Integrated genetic and epigenetic analysis defines novel molecular subgroups in rhabdomyosarcoma

**DOI:** 10.1038/ncomms8557

**Published:** 2015-07-03

**Authors:** Masafumi Seki, Riki Nishimura, Kenichi Yoshida, Teppei Shimamura, Yuichi Shiraishi, Yusuke Sato, Motohiro Kato, Kenichi Chiba, Hiroko Tanaka, Noriko Hoshino, Genta Nagae, Yusuke Shiozawa, Yusuke Okuno, Hajime Hosoi, Yukichi Tanaka, Hajime Okita, Mitsuru Miyachi, Ryota Souzaki, Tomoaki Taguchi, Katsuyoshi Koh, Ryoji Hanada, Keisuke Kato, Yuko Nomura, Masaharu Akiyama, Akira Oka, Takashi Igarashi, Satoru Miyano, Hiroyuki Aburatani, Yasuhide Hayashi, Seishi Ogawa, Junko Takita

**Affiliations:** 1 Department of Pediatrics, Graduate School of Medicine, The University of Tokyo, Tokyo 113-8655, Japan; 2 Department of Pathology and Tumor Biology, Graduate School of Medicine, Kyoto University, Kyoto 606-8501, Japan; 3 Cancer Genomics Project, Graduate School of Medicine, The University of Tokyo, Tokyo, 113-8655, Japan; 4 Laboratory of DNA Information Analysis, Human Genome Center, Institute of Medical Science, The University of Tokyo, Tokyo 108-8639, Japan; 5 Division of Systems Biology, Nagoya University Graduate School of Medicine, Nagoya 466-8550, Japan; 6 Department of Cell Therapy and Transplantation Medicine, The University of Tokyo, Tokyo 113-8655, Japan; 7 Department of Hematology/Oncology, Saitama Children's Medical Center, Saitama 339-8551, Japan; 8 Laboratory of Sequence Data Analysis, Human Genome Center, Institute of Medical Science, The University of Tokyo, Tokyo 108-8639, Japan; 9 Department of Pediatric Surgery, Graduate School of Medicine, The University of Tokyo, Tokyo, 113-8655, Japan; 10 Genome Science Division, Research Center for Advanced Science and Technology, The University of Tokyo, Tokyo 153-8904, Japan; 11 Department of Pediatrics, Nagoya University Graduate School of Medicine, Nagoya 466-8550, Japan; 12 Department of Pediatrics, Kyoto Prefectural University of Medicine, Graduate School of Medical Science, Kyoto 602-8566, Japan; 13 Department of Pathology, Kanagawa Children's Medical Center, Yokohama 232-8555, Japan; 14 Molecular Pathology Laboratory, Department of Pediatric Hematology and Oncology Research, National Research Institute for Child Health and Development, Tokyo 157-8535, Japan; 15 Department of Pediatric Surgery, Reproductive and Developmental Medicine, Faculty of Medical Sciences, Kyushu University, Fukuoka 812-8582, Japan; 16 Division of Pediatric Hematology and Oncology, Ibaraki Children's Hospital, Mito 311-4145, Japan; 17 Department of Pediatrics, School of Medicine, Fukuoka University, Fukuoka 814-0180, Japan; 18 Department of Pediatrics, The Jikei University School of Medicine, Tokyo 105-8471, Japan; 19 National Center for Child Health and Development, Tokyo 157-8535, Japan; 20 Department of Hematology/Oncology, Gunma Children's Medical Center, Shibukawa, Gunma, 377-8577, Japan

## Abstract

Rhabdomyosarcoma (RMS) is the most common soft-tissue sarcoma in childhood. Here we studied 60 RMSs using whole-exome/-transcriptome sequencing, copy number (CN) and DNA methylome analyses to unravel the genetic/epigenetic basis of RMS. On the basis of methylation patterns, RMS is clustered into four distinct subtypes, which exhibits remarkable correlation with mutation/CN profiles, histological phenotypes and clinical behaviours. A1 and A2 subtypes, especially A1, largely correspond to alveolar histology with frequent *PAX3/7* fusions and alterations in cell cycle regulators. In contrast, mostly showing embryonal histology, both E1 and E2 subtypes are characterized by high frequency of CN alterations and/or allelic imbalances, *FGFR4/RAS/AKT* pathway mutations and *PTEN* mutations/methylation and in E2, also by p53 inactivation. Despite the better prognosis of embryonal RMS, patients in the E2 are likely to have a poor prognosis. Our results highlight the close relationships of the methylation status and gene mutations with the biological behaviour in RMS.

## Abstract (web summary)

Rhabdomyosarcoma is a common childhood soft-tissue cancer. Here Seki and Nishimura analyse the exome, transcriptome, copy number and DNA methylome of 60 sarcomas and identify distinct methylation subgroups associated with genetic and clinical features.

## Graphical abstract

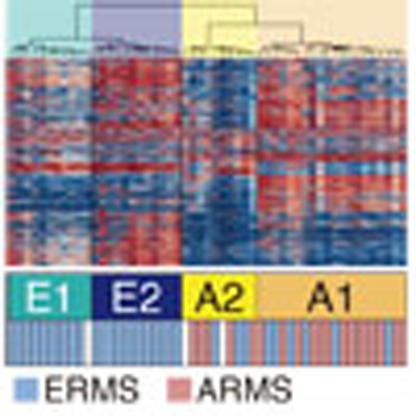

Rhabdomyosarcoma (RMS), a highly aggressive soft-tissue sarcoma, typically affects children, and it is classified into two major subtypes having alveolar (ARMS) and embryonal (ERMS) histologies^1^,^2^. ARMS usually carries specific chromosomal translocations that result in *PAX3*– or *PAX7*–*FOXO1* fusion genes, whereas ERMS commonly harbours loss of heterozygosity at 11p15.5 and gains of chromosomes 2, 8, and 12 in varying combinations^3^,^4^. Despite aggressive multimodal therapies, the prognosis of high-risk RMS patients has not been substantially improved, with a 5-year overall survival rate being <20–30%^5^, which prompts a need for new therapeutic strategies targeting molecular pathways that are relevant to the pathogenesis of RMS. In this point of view, recent sequencing studies have revealed a number of recurrent mutational targets of RMS, including multiple components of the *FGFR4/RAS/AKT* pathway, *FBXW7*, *CTNNB1* and *BCOR*^6^,^7^. However, the relatively low number of mutations in RMS (12.3/tumor sample^7^) suggests the involvement of other mechanisms, such as epigenetic alterations, which have not been disclosed in the previous studies^6^,^7^,^8^. To address these issues, we conducted an integrated molecular study in which a cohort of 60 RMSs cases was investigated for somatic mutations, CN alterations, and DNA methylomes using whole-exome/targeted deep sequencing, Single-nucleotide polymorphism (SNP) array-based CN analysis, and Infinium 450 K arrays, respectively. RNA sequencing was also performed for the selected tumours for which high-quality RNA was available (that is, RNA integrity number >6.0). Here we identify novel methylation clusters that exhibit distinct genetic abnormalities, histological subtypes and clinical behaviours, suggesting that aberrant DNA methylation along with genetic alterations is likely to play key roles in the pathogenesis of RMS.

## Results

### Sequencing and CN analyses of RMS

We first sequenced the exome of 16 paired tumours/normal samples of which 3 were also analysed for relapsed (*n*=2) and metastatic (*n*=1) samples (Supplementary Data 1). The mean coverage was 123 × , with which 88% of the target exome sequences were analysed at a depth of more than 20 × (Supplementary Fig. 1). Among 690-candidate somatic changes detected by our pipeline called Genomon (http://genomon.hgc.jp/exome/en/index.html), 604 in 531 genes (88%) were validated by deep sequencing (Supplementary Data 2). The mean numbers of mutation in primary, metastatic, and relapsed tumours were 24.0, 43.3 and 42.0, respectively. Thus, the mutation rate was slightly higher in relapsed/metastatic tumours than in primary tumours, although not statistically significant (primary versus metastasis, *t*-test *P*=0.20 and primary versus relapsed, *P*=0.13; Supplementary Fig. 2). An excessively high number of somatic mutations was present in one case (RMS_001) in which an *MBD4* gene mutation was implicated in defective DNA repair^9^ (Supplementary Fig. 2). As observed in other cancers, mutations were predominated by C>T/G>A transitions compared with other transitions or transversions^10^ (Supplementary Fig. 3).

**Figure 1:**
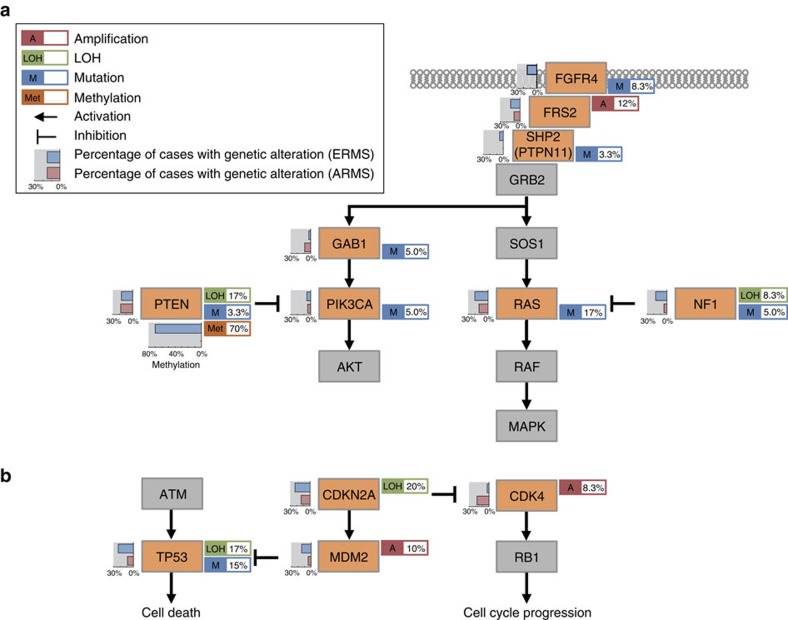
Significantly altered pathways in rhabdomyosarcoma. The *FGFR4/RAS/AKT* pathway (**a**), cell cycle, and p53 signalling (**b**) are frequently altered. Genes with genetic alterations are coloured in light orange. The types of alterations and their frequency in the population are indicated on the right side of each gene. The percentages of cases with alterations including copy-number alterations and/or gene mutations detected in embryonal and alveolar subgroups are separately indicated on the left side of each gene. *PTEN* methylation is also indicated below the gene name and coloured in orange.

Among the 531 mutated genes, only 18 were recurrently mutated (Table 1), which not only included known mutational targets in RMS, such as *TP53*, *BCOR*, *KRAS* and other genes in the *FGFR4/RAS/AKT* pathway, but also involved in previously unreported genes, including *ROBO1* and additional *FGFR4/RAS/AKT* pathway genes^6^,^7^,^8^,^11^, such as *GAB1* and *PTEN* (Table 1). Thus, to validate the initial observation in the discovery samples and investigate the impact of these mutations on the pathogenesis and clinical outcomes of RMS, we performed follow-up deep sequencing for 14 putative driver genes in the entire cohort of 60 RMS cases including the 16 discovery cases (Supplementary Data 3). Overall, 56 mutations were found in the 14 genes (Table 2, Supplementary Data 4, Supplementary Fig. 4). The most frequently mutated genes were *TP53* (9/60; 15.0%) and *FGFR4/RAS/AKT* pathway genes (24/60; 40%), which were predominantly detected in ERMS tumours^7^ (Fig. 1, Table 2). Among *FGFR4/RAS/AKT* pathway mutations, RAS pathway genes were mutated in 15 cases, in which all mutations in *NRAS/KRAS/HRAS* (*n*=10) and *PTPN11* (*n*=2) occurred in hot-spot amino acids involved in gene activation, whereas three of four *NF1* mutations were frameshift indels resulting in premature truncation of the protein. Five of six *FGFR4* mutations affected highly conserved amino acids within the kinase domain of which four were previously reported activating mutations^11^, N535K and V550L. Additional four mutations involved *PIK3CA*, which have previously been reported in other cancers, are thought to be activating in nature^12^,^13^,^14^. Other commonly mutated genes were *BCOR* and *ARID1A*. All *BCOR* mutations and four of six *ARID1A* mutations were frameshift or nonsense mutations, suggesting the importance of inactivation of these genes in the pathogenesis of RMS.

**Figure 2:**
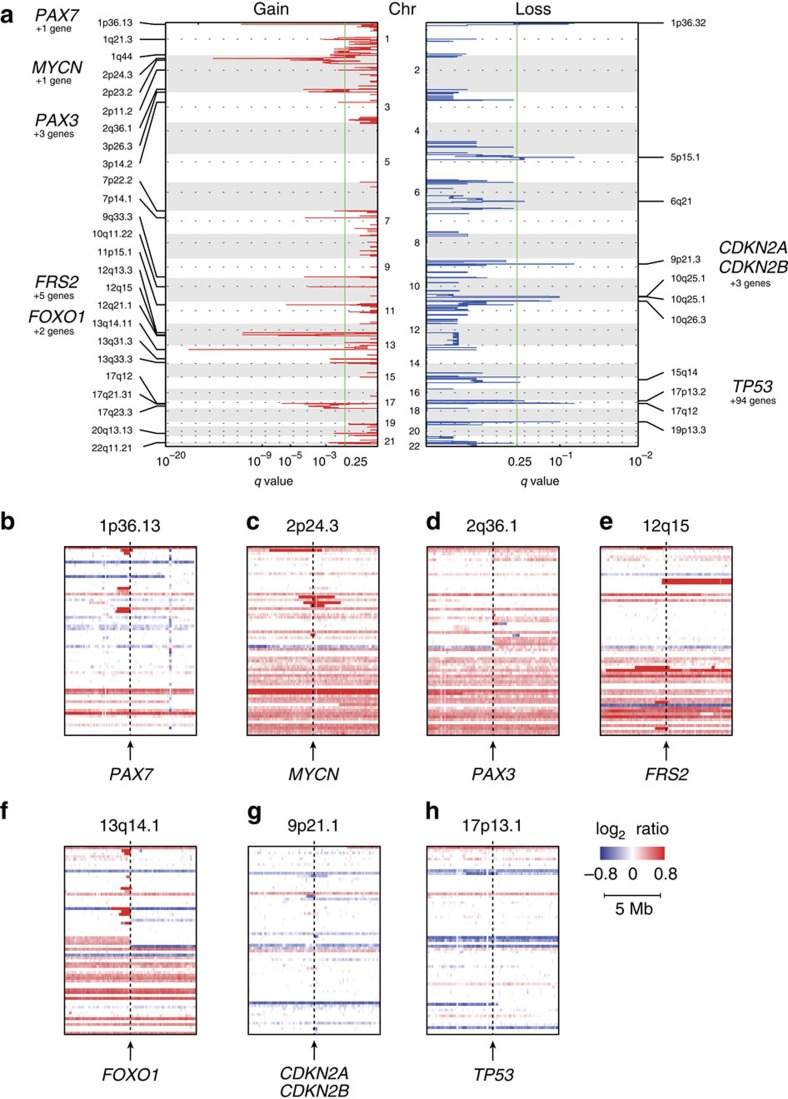
Significant copy-number (CN) alterations detected in rhabdomyosarcoma. (**a**) Statistically significant CN gains and losses detected by the GISTIC algorithm are shown in the left and right boxes, respectively. For each *q*-value peak, the putative gene targets are listed. A dashed line represents the centromere of each chromosome. Red and blue lines indicate the *q*-value for gains and losses, respectively. (**b**–**h**) Heatmaps of significant CN alterations are shown for gene targets at 1p36.13 (**b**), 2p24.3 (**c**), 2q36.1 (**d**), 12q15 (**e**), 13q14.1 (**f**), 9p21.1 (**g**) and 17p13.1 (**h**).

A number of genes and pathways were also recurrently affected by CN alterations and thought to be implicated in deregulated *FGFR4/RAS/AKT* signalling (focal amplification of *FRS2* at 12q15; 12%) and cell cycle regulation (focal amplification of *MYCN* at 2p24.3, loss of *CDKN2A/B* at 9p21, *TP53* at 17p13.2, and *MDM2* at 12q15; Figs 1 and 2, Supplementary Fig. 5). Other genes displaying significant CN alterations included *ALK* (2p23.2) and *STAT6* (12q13.3) (Supplementary Data 5 and 6). As previously reported, the ARMS-related fusion genes *PAX3*/*7* (*n*=6) and *FOXO1* (*n*=6) were frequently accompanied by local amplifications^15^,^16^.

### Transcriptome analysis of RMS

Fusion transcripts were investigated by RNA sequencing in eight cases of RMS, including five ERMS and three ARMS cases (Supplementary Data 1). In total, we identified 22 fusion transcripts, of which three were predicted to be in-frame, whereas the remaining 19 were out-of-frame fusions. Expression of fusion transcripts was confirmed by reverse transcription-PCR (RT–PCR) for 2 in-frame and 10 out-of-frame fusions (Supplementary Data 7), but no recurrent fusions were identified, except for the *PAX3*–*FOXO1* fusion found in two cases of ARMS. The *NSD1*–*ZNF346* fusion that was previously identified in an ERMS cell line^6^, was found in a case of ERMS. However, the *NSD1*–*ZNF346* fusion detected in our study was out-of-frame and thus functional significance of this fusion transcript is still elusive.

### Novel clusters identified by DNA methylation analysis

**Figure 3:**
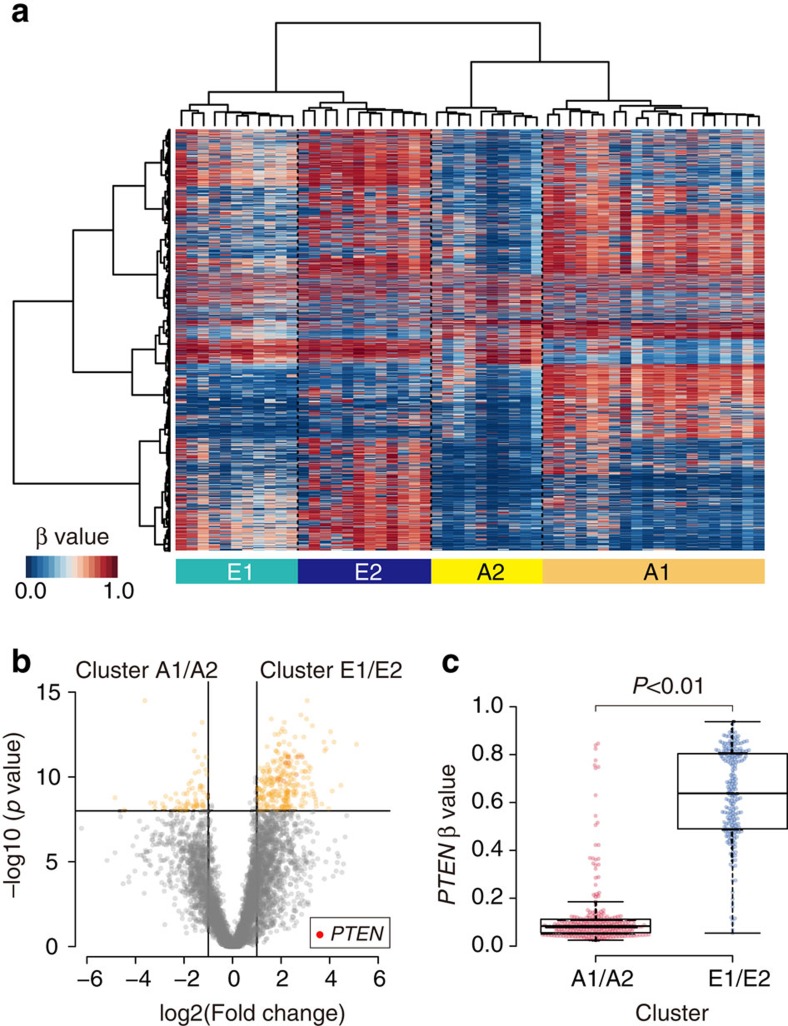
Hierarchical clustering of DNA methylation profiles and significant *PTEN* hypermethylation in cluster E1/E2. (**a**) The heatmap shows the DNA methylation profiles of 53 rhabdomyosarcoma (RMS) tumours based on unsupervised hierarchical clustering. Tumours were hierarchically clustered into four subgroups: E1, E2, A1 and A2. Clusters E1 and E2 were composed almost exclusively of embryonal RMS (95.5%), whereas all alveolar RMS were classified into clusters A1/A2. Red, high methylation; blue, low methylation. (**b**) Volcano plot comparing the number of significantly hypermethylated probes between clusters E1/E2 and A1/A2. Significantly hypermethylated probes showing >2-fold change are coloured in orange (*P*<1.0 × 10^−8^, Wilcoxon's rank-sum test). Red dots represent significantly hypermethylated probes in the promoter regions of *PTEN* in cluster E. (**c**) Difference in *PTEN* methylation intensities between clusters E1/E2 and A1/A2. Significantly higher methylation intensities were observed in cluster E1/E2 than in cluster A1/A2. The *P*-value was calculated using the Wilcoxon rank-sum test. β-values represent significantly hypermethylated eight promoter-associated probes (red dots in the Volcano plot) of PTEN. For the box and whisker plots, the bottom and top of the box are the first and third quartiles, the line inside the box is the median and the whiskers extend up to 1.5 times the interquartile range.

To further explore the molecular basis of RMS, we investigated genome-wide DNA methylation in 53 RMS tumours using Infinium HumanMethylation450 BeadChip (Illumina). DNA methylation profiling based on unsupervised hierarchical clustering identified four unique clusters having distinct methylation signatures (Fig. 3a), and the microarray data were validated by bisulfite sequencing for selected probes (*n*=160; Supplementary Fig. 6). Remarkably, we found that these clusters correlated with histological subtypes, genetic abnormalities and clinical outcomes. Two clusters, E1 and E2, were composed almost exclusively of ERMS (95.5%; Fig. 3a), whereas all cases of ARMS were grouped into the two remaining clusters A1 and A2 (Fig. 3a). Accordingly, all tumours positive for *PAX3*–*FOXO1* or *PAX7*–*FOXO1* fusions were grouped into the A1/A2 clusters, although the separation between A1 and A2 did not coincide with the presence or absence of fusions (Fig. 3a). In our analysis, 29 genes were significantly hypermethylated in the E1/E2 clusters compared with the A1/A2 clusters. On the other hand, only 10 genes were significantly hypermethylated in the A1/A2 clusters compared with those in the E1/E2 cluster (Fig. 3b; Supplementary Fig. 7; Supplementary Data 8). Among these, the largest extent of promoter methylation was observed in *PTEN* in the clusters E1/E2 and *GATA4* in the clusters A1/A2. Of note, we found an extremely high frequency of *PTEN* hypermethylation in E/E2 tumours (20 of 22; 90.9%), whereas only two of 28 (7.1%) in A1/A2 tumours showed *PTEN* hypermethylation (Fig. 3c). Subsequent quantitative RT–PCR analysis revealed that *PTEN* hypermethylation was significantly associated with the absence of *PTEN* expression, suggesting that epigenetic silencing of *PTEN* is a representative mechanism of E1/E2 tumours (*P*=0.045; Supplementary Fig. 8). *GATA4* encodes a member of the GATA family of transcription factors^17^,^18^ and has been shown to be methylated in a subset of RMS cases^19^. However, because *GATA4* expression is limited to the heart, testis and ovary, significance of methylation in RMS pathology is still unclear. We also found 23 genes significantly hypermethylated in E2 compared with E1 and 25 genes in A1 to A2 (Supplementary Fig. 9, Supplementary Data 9 and 10). Although these findings should be validated in independent samples, methylation of these genes could be potentially used for discriminating these subtypes.

### Characteristics of the methylation subgroups in RMS

To obtain a better understanding of DNA methylation in each subgroup, we further performed Ingenuity pathway analysis (http://www.ingenuity.com/) using probe lists of E1/E2 versus A1/A2, E1 versus E2 and A1 versus A2 (Supplementary Data 8–10). However, relevant cancer-associated pathways in the pathogenesis of RMS were not detected in the A1/A2 and E1/E2 subgroups (Supplementary Data 11 and 12). A functional annotation named ‘abnormal morphology of muscle,' containing *DYSF*, *ELN*, *OTX2* and *TH*, was detected as a significantly hypermethylated component in A1 compared with A2 (*P*=6.2 × 10^−4^; Supplementary Data 13–16). In addition, the promoter of *P4HTM,* the family of prolyl 4-hydroxylases, was significantly hypermethylated in E2 compared with E1 (*P*=0.035; Supplementary Fig. 9). Intriguingly, *P4HTM* hypermethylation was also reported in a previous study regarding DNA promoter methylation in 10 RMS tumours^19^. However, the biological significance of these genes in the pathogenesis of RMS is still unclear.

**Figure 4:**
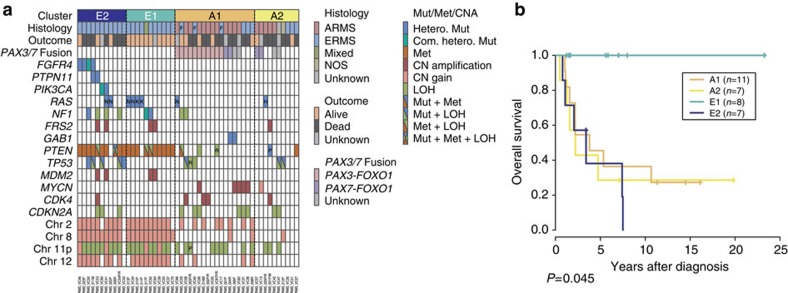
Correlations between DNA methylation clusters and additional parameters. (**a**) Integrated view of DNA methylation clusters combined with histology, outcome, fusion status, gene alterations and copy-number changes. The horizontal axis represents each tumor. ARMS, alveolar rhabdomyosarcoma; ERMS, embryonal rhabdomyosarcoma; NOS, not otherwise specified; Mut, mutation; Met, methylation; CNA, copy-number alteration; Hetero., heterozygous; Com. Hetero., compound heterozygous mutation; CN, copy number; LOH, loss of heterozygosity; N, *NRAS*; K, *KRAS*; H, *HRAS*; P, detected in primary sample alone; R, Detected in relapse sample alone; F, *PAX3–FOXO1* fusion-positive ERMS. (**b**) Kaplan–Meier curves of overall survival for each DNA methylation cluster. The *P*-value of the log-rank test is shown.

Next, we analysed the genetic and clinical characteristics of each methylation subgroup. E1 tumours showed a lower mutation rate (mean, 20.0/sample) than E2 tumours (mean, 45.0/sample), although not statistically significant (*P*=0.084). Compared with the A1/A2 clusters, the E1/E2 clusters were characterized by high frequencies of multiple chromosomal CN changes including gains of chromosomes 2, 8 or 12 (*P*=5.2 × 10^−4^). In addition, the mutation frequency of the *FGFR4/RAS/AKT* pathway was much higher in the E1/E2 clusters than in the A1/A2 clusters (*P*=4.6 × 10^−4^), and these mutations were predominantly observed in the E2 cluster (54.5% in E1 and 81.8% in E2 versus 17.9% in A1/A2; Fig. 4; Table 2). Among the *FGFR4/RAS/AKT* pathway mutations, mutations in *FGFR4*, *PTPTN11* and *PIK3CA* frequently occurred in E2 compared with E1 (*P*=1.0 × 10^−3^). Furthermore, E2 tumours had significantly frequent *TP53* mutations (45.5%) compared with E1 tumours (0%; *P*=0.035). In contrast, mutations and CN alterations affecting cell cycle regulators, such as *MYCN*, *CDK4* and *CDKN2A/B*, except for *MDM2*, were particularly enriched in the A1/A2 clusters compared with the E1/E2 clusters (*P*=0.016), suggesting that these genes may have driver roles in A1/A2 tumours.

Finally, patients in the E2 cluster displayed significantly worse overall survival than those in the E1 cluster, regardless of stage, age and the site of tumour involvement (*P*=0.045; Fig. 4b). In addition, among commonly mutated genes and pathways, only *TP53* mutations but not *FGFR4/RAS/AKT* pathway mutations (including *FGFR4*, *PTPN11*, *PIK3CA* and *PTEN* mutations) significantly affected the overall survival of E1/E2 patients (*P*=2.9 × 10^−3^). However, the impact of the E1/E2 classification on overall survival was more prominent than that of the *TP53* mutations (Supplementary Fig. 10). These results suggest that the methylation status that defines the E1/E2 clusters might be a predictor of overall survival, independent of gene mutations.

## Discussion

Our sequencing screen identified both previously well-recognized gene mutations including *FGFR4/RAS/AKT* pathway mutations and novel recurrently mutated genes, such as *PTEN*, *GAB1* and *ROBO*. Most *FGFR4/RAS/AKT* pathway alterations, excluding *GAB1,* were predominantly found in ERMS (Fig. 1), suggesting that deregulated *FGFR4/RAS/AKT* signalling plays an important role in the pathogenesis of ERMS. Because the *PTEN* missense mutation affecting G129 has been reported in several cancers^20^,^21^ and is thought to abrogate most PTEN activity, the *PTEN* G129R mutation detected in RMS should have an oncogenic effect. The allele frequency of the *PTEN* A120P mutation was 0.84, and in accordance with this finding, the SNP array analysis disclosed uniparental disomy involving the *PTEN* locus by which the mutated allele was duplicated. Thus, the A120P substitution is likely to be an oncogenic mutation rather than a non-functional SNP. Although somatic mutations in *GAB1* and *ROBO1* have been reported in several cancers, the role of these genes in the RMS pathogenesis is unknown^22^,^23^,^24^.

Probably, the most significant finding in the current study would be the identification of the novel methylation clusters, which tightly correlated with genetic abnormalities, histological subtypes and clinical behaviours, underscoring the importance of integrated molecular analyses. Importantly, this finding revealed an otherwise unrecognizable subset of ERMS that showed a poor prognosis (E2 cluster) for which intensification or novel therapeutic approaches need to be considered. We also found an extremely high frequency of epigenetic silencing of *PTEN* in ERMS. *PTEN* is one of the most frequently mutated tumour suppressors in human cancers and is also essential for embryonic development^25^. PTEN modulates G1 cell cycle progression by negatively regulating the *FGFR4*/*PI3K/AKT* axis. Thus, the finding that *PTEN* methylation/mutation is highly specific to and frequent in the E1/E2 tumours indicates that *PTEN* may serve as a diagnostic marker to identify those patients for which inhibition of the *FGFR4/PI3K/AKT* axis could have a key therapeutic role. Finally, the biological/genetic basis for these distinct methylation clusters, nevertheless, is currently unknown and it should be addressed in future studies. Since our single cohort in the current study is very limited, to provide an adequate assessment of molecular subgroups, more large number of independent cohorts should be evaluated.

## Methods

### Samples

This study cohort comprised 66 tumours from 60 cases with RMS (22 ARMS, 35 ERMS, one mixed type, one RMS, not otherwise specified, and one unknown histology. Matched normal specimens (mononuclear cells from peripheral blood or bone marrow at diagnosis or in remission) were available in 16 cases, which were subjected to whole-exome sequencing. In three ARMS cases, samples from both primary and recurrent or metastatic tumours were available, which were analysed by whole-exome sequencing. Detailed information on subjects and samples is provided in Supplementary Data 1 and 3. To generate sufficient DNA templates of tumour and germline DNA in a subset of cases in which enough samples were not available, whole-genome amplification was performed using the REPLI-g kit (Qiagen). The amplified DNAs were used in part for targeted deep sequencing to validate individual candidate mutations detected in whole-exome sequencing; to analyse all coding sequences of possible candidate genes in the validation cohort. Written informed consent for research use was obtained from patients' parents, and this study was approved by the Human Genome, Gene Analysis Research Ethics Committee of the University of Tokyo.

### Whole-exome sequencing

In 19 samples from 16 cases, whole-exome capture libraries were constructed from tumour and matched normal DNA using Agilent SureSelect Human All Exon 50 Mb, V4 or V5 (Agilent Technologies) according to the manufacturer's protocol. Enriched exome libraries were sequenced with the Hiseq 2,000 platform (Illumina). For identifying candidates for somatic mutations from exome-sequencing data, the EBcall (Empirical Bayesian mutation calling)^26^ algorism was used. This algorism discriminates somatic mutations from sequencing errors based on an empirical Bayesian method using sequencing data of multiple non-paired germline samples.

### RNA sequencing

In eight tumour samples from our RMS which high-quality RNA (that is, RNA integrity number >6.0) was available, RNA sequencing was performed using Hiseq 2,000. RNA sequencing libraries were prepared using the Truseq RNA Sample Preparation kit (Illumina). Candidates of fusion transcripts were identified using Genomon-fusion. All candidates of gene fusion, which represented more than two paired-reads were subjected to confirm by RT–PCR-based Sanger sequencing. The primer sets used for RT–PCR analysis were listed in Supplementary Data 7.

### Validation of putative somatic variants

To validate putative genomic variants detected in whole-exome sequencing, targeted deep sequencing was performed^27^. Regions encompassing possible variations were amplified with *Not*I-tagged primers. Pooled PCR amplicons were digested with *Not*I, followed by ligation with T4 DNA ligase. Ligated PCR amplicons were sonicated into fragments of an average size of 200 bp using a Covaris sonicator. After being enriched and multiplexed, the prepared libraries were subjected to deep sequencing using Illumina Hiseq 2,000 or Miseq with a 100-bp or 150-bp pair-end reads option. Both tumour and germline DNA were examined to confirm somatic variations. Selective variants of which PCR primers could be constructed by Primer 3^28^,^29^ were subjected to deep sequencing for validation. The validated mutations are listed in Supplementary Data 2.

### Evaluation of mutation candidates

For 14 candidate genes, including recurrent mutated genes in exome sequencing (*TP53*, *BCOR*, *ARID1A*, *GAB1*, *ROBO1*, *PTEN* and *KRAS*), and the associated genes (*FGFR4*, *PIK3CA*, *HRAS, NRAS, PTPN11, NF1* and *FBXW7*), PCR-based deep sequencing of the candidate genes was performed in 66 samples using Hiseq 2000 or Miseq (Illumina). *Not*I-tagged primers (Supplementary Data 17) were used to generate each PCR amplicon. Selected exons were sequenced for *FGFR4, PIK3CA and PTPN11*. Sample preparation, data processing and variant detection were performed as described above in the methods section of Validation of putative somatic variants.

### DNA methylation analysis

In 53 RMS samples from 50 cases, comprehensive DNA methylation analysis was performed with the Infinium HumanMethylation450 BeadChip (Illumina) according to the manufacturer's protocol. Beta-values were converted to *M*-values^30^, then pcaMethods bioconductor R package was used to impute incomplete data. *M*-values were converted to beta-values again and imputed beta-values were used for further analyse. To determine the DNA methylation profiles, the following steps were adopted to select probes for unsupervised clustering analysis. We first removed probes that were designed for sequences on the X and Y chromosomes. Second, we selected probes with variance ranked in the top 1% of the remaining probes. We then performed unsupervised hierarchical clustering with 4,708 probes, identifying 4 distinct clusters. To detect methylation status compared with normal skeletal muscles, published data of the same 450 k platform was used^31^. Wilcoxon rank-sum test was performed to select differentially methylated probes. To detect differentially methylated genes for each sample, multiplicative decomposition model of gene methylation was adopted. Let *x*_ijk_ be the beta value at probe location *j* within 1,500 bps of the transcription start site of gene *k* for RMS sample *i*, *c*_jk_ be the average beta value at the same location of the same gene for 48 normal skeletal muscle samples, *n* be the number of samples, *g* be the number of genes, and *p*_k_ be the number of probes within 1,500 bps of transcription start site of gene *k*. For each gene *k*, we consider a following multiplicative model of the two-way table of gene methylation changes:


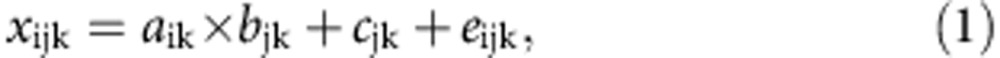


where *e*_ijk_ is an observation error, *a*_ik_ is the methylation pattern depending on sample *i*, and *b*_jk_ is the methylation pattern depending on probe location *j*, respectively. The parameters *a*_ik_, *i*=1,…,*n* and *b*_jk_, *j*=1,…,*p*_k_ are estimated by Bayesian principal component analysis^32^. The signs of *a*_ik_, *i*=1,…,*n* are chosen so that they are proportional to the average beta value of *p*_k_ locations of gene *k*. The *z*-score to see if *a*_ik_ is significantly larger or smaller than those of the normal skeletal muscles is then calculated by:


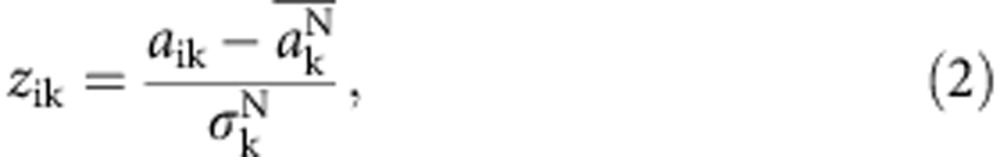


where 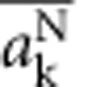 and 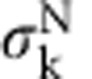 are the average and s.d. of the sample-dependent methylation patterns for the normal skeletal muscles, respectively.

For a given threshold *T*, we can declare the methylation status of gene *k* for sample *i* to be an aberration compared with that of normal skeletal muscles if |*z*_ik_|>*T*. Here identifying aberrations can be thought of as a multiple-testing problem where we are testing the following hypothesis for each gene within each sample:


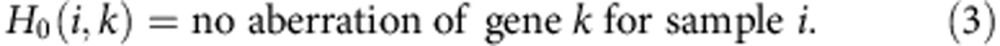


An estimator of the false discovery rate^33^ for threshold *T* can be calculated by


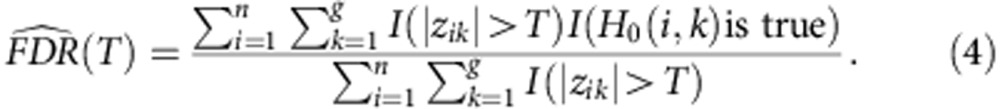


The threshold *T* is then chosen so that 
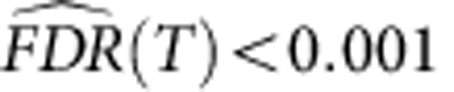
.

### Quantitative RT–PCR

Quantitative RT–PCR for PTEN was performed in 19 samples from which enough RNA was available. RNA (200 ng) extracted from the fresh-frozen tumours were subjected to reverse transcription using the SuperScript VILO MasterMix (Invitrogen) according to the manufacturer's protocol. Quantitative mRNA expression levels were measured using the QuantiTect SYBR Green PCR kit (Qiagen, Tokyo, Japan) with an iCycler iQ real-time PCR detection system (Bio-Rad). The optional thermal-cycling condition was as follows: 40 cycles of a two-step PCR (95 °C for 15 s, 58 °C for 60 s) after the initial denaturation (95 °C for 10 min). For the purpose of normalization, relative expression levels were calculated by dividing the expression level of the respective gene by that of GAPDH^34^. Primers used for quantitative RT–PCR are listed in Supplementary Data 18.

### Bisulfite conversion and bisulfite sequencing of *PTEN*

To confirm *PTEN* methylation, bisulfite sequencing using nested primers was performed. Genomic DNA (500 ng) was bisulfite-modified using the EpiTect Plus DNA Bisulfite Kit (Qiagen) according to the manufacturer's instructions. Primer sequences for PCR amplicons are listed in Supplementary Data 18. Complete bisulfite modification was confirmed by bisulfite sequence analysis of the replicated normal DNA.

### Bisulfite sequencing for validation of methylation array

To validate the result of DNA methylation array, targeted bisulfite deep sequencing was performed. Five samples among each cluster were randomly selected for validation. As positive controls, two samples of replicated normal DNA were also analysed by bisulfite sequencing. Genomic DNA (1,000 ng) was bisulfite modified using the EpiTect Plus DNA Bisulfite Kit (Qiagen) according to the manufacturer's instructions. Among extracted 266 probes (E1/E2 versus A1/A2, E1 versus E2 and A1 versus A2), we selected probes to validate, which recurrently detected in three or more. Finally, 160 probes were subjected to target deep sequencing. Bisulfite PCR was performed in 22 samples (20 tumours and two controls) with *Not*I-tagged primes as described above. For data analysis, the same method of targeted deep sequencing was used, with modified mapping to bisulfite converted reference genome. Methylated cytosine was calculated as variant allele frequency. Validated probes were listed in Supplementary Data 19. The Spearman correlation coefficient was used to compare beta-values and methylated allele frequencies.

### Pathway analysis

Ingenuity pathway analysis (http://www.ingenuity.com/) was performed for extracted genes listed in Supplementary Data 8–10.

### SNP array analysis

DNA extracted from RMS samples was subjected to SNP array analysis using Affymetrix GeneChip 250K *Nsp* or CytoScan HD (Affymetrix) according to the manufacturer's protocol. Details regarding the use of these platforms are listed in Supplementary Data 1 and 3. CNAG/AsCNAR software was used for subsequent informatics analysis for SNP array data, which enables accurate detection of allelic status without paired normal DNA, even in the presence of up to 70–80% normal cell contamination^35^,^36^. Significant focal CN alterations were identified using GISTIC 2.0^37^ for 250 k array data. The array data have been partially published in the previous our paper^3^.

### Analyses of *PAX* gene fusions

The status of the *PAX3/7*–*FOXO1* fusion gene was examined by RT–PCR followed by Sanger sequencing in 34 tumours for which RNA or cDNA was available. The primers and the PCR condition are listed in Supplementary Data 20.

### Statistical analysis

Fisher's exact test was used to evaluate the differences in chromosomal CN changes between ARMS and ERMS or the correlation between CN changes and prognosis in each subgroup. The numbers of mutations or CN changes were compared by *t*-test.

## Additional information

**Accession codes:** Whole-exome sequence data and the methylation microarray data has been deposited in the European Nucleotide Archive, hosted by the European Bioinformatics Institute, under the accession code EGAS00001000884. The SNP array data have been deposited in the Gene Expression Omnibus under accession number GSE41263 and GSE63891.

**How to cite this article:** Seki, M. *et al.* Integrated genetic and epigenetic analysis defines novel molecular subgroups in rhabdomyosarcoma. *Nat. Commun.* 6:7557 doi: 10.1038/ncomms8557 (2015).

## Supplementary Material

Supplementary Figures Supplementary Figures 1-10

Supplementary Data 1 Patient characteristics of RMS cases analyzed by next generation sequencing.

Supplementary Data 2 List of detected mutations by whole exome sequencing.

Supplementary Data 3 Patient characteristics of validation cohort.

Supplementary Data 4 List of detected mutations by targeted deep sequencing.

Supplementary Data 5 Significant gain regions detected by GISTIC analysis.

Supplementary Data 6 Significant loss regions detected by GISTIC analysis.

Supplementary Data 7 Validated fusion transcripts detected in RNA sequencing.

Supplementary Data 8 Differentially methylated probes between cluster E1/E2 and A1/A2.

Supplementary Data 9 Differentially methylated probes between cluster E1 and E2.

Supplementary Data 10 Differentially methylated probes between cluster A1 and A2.

Supplementary Data 11 Pathway analysis for gene list of E1/E2 vs. A1/A2.

Supplementary Data 12 Functions annotation analysis for gene list of E1/E2 vs. A1/A2.

Supplementary Data 13 Pathway analysis for gene list of E1 vs. E2.

Supplementary Data 14 Functions annotation analysis for gene list of E1 vs. E2.

Supplementary Data 15 Pathway analysis for gene list of A1 vs. A2.

Supplementary Data 16 Functions annotation analysis for gene list of A1 vs. A2.

Supplementary Data 17 Primer sets for targeted deep sequencing.

Supplementary Data 18 Primer sets for nested PCR of bisulfite sequencing and RT-PCR in PTEN.

Supplementary Data 19 Selected probes for validation of methylation array and primer sequences for bisulfite targeted deep sequencing.

Supplementary Data 20 Primer sequences for RT-PCR.

## Figures and Tables

**Table 1:** Recurrent mutations in rhabdomyosarcoma cases detected by whole-exome sequencing.

**Gene**	**Location**	***N***	**Amino-acid change**	**Histology**	**Sample ID**
*TP53*	17p	2	G245S	ERMS	RMS_011M
			C176G	NOS	RMS_012P
*GAB1*	4q	2	S614R	ARMS	RMS_004R
			N233I	ARMS	RMS_006P
*KRAS*	12p	2	K117N	ERMS	RMS_014P
			A146T	ERMS	RMS_015P
*PTEN*	10q	2	G129R	ARMS	RMS_001P
			A120P	ERMS	RMS_009R
*ARID1A*	1p	2	G1847fs	ARMS	RMS_003R
			K1007_I1008fs	ARMS	RMS_011M
*BCOR*	Xp	2	S1243fs	ERMS	RMS_014P
			E712fs	Mixed	RMS_017P
*ROBO1*	3p	2	E1113Q	ARMS	RMS_005P
			Y777C	ARMS	RMS_007P
*AKAP9*	7q	2	G2652V	ARMS	RMS_001M
			K2673N	ERMS	RMS_009R
*DNAH5*	5p	2	S2920I	ERMS	RMS_011M
			R3096Q, D3271N	ERMS	RMS_008R
*FREM2*	13q	2	R1515H	Mixed	RMS_017P
			Q1574L	ERMS	RMS_008R
*C15orf2*	15q	2	G917A	ARMS	RMS_003P
			V1147L	ERMS	RMS_009R
*KIF21A*	12q	2	E766V	Mixed	RMS_017P
			L956P	ERMS	RMS_009R
*NEB*	2q	2	E637Q	ARMS	RMS_001P
			D1595Y	ARMS	RMS_003P
*PTPRO*	12p	2	R67T	ARMS	RMS_002R
			N644S	Mixed	RMS_017P
*COL5A2*	2q	2	G203V	ARMS	RMS_001P
			G756S	ARMS	RMS_002P
*PXDNL*	8q	2	T877M	ARMS	RMS_004R
			R1225Q	ARMS	RMS_016M
*NLRC5*	16q	2	R341W	ARMS	RMS_003P
			Q1169H	ARMS	RMS_001P
*TTN*	2q	4	L18955Q, L18955I	ARMS	RMS_001M
			G17007A	ARMS	RMS_004R
			E5800A	ERMS	RMS_009P
			Q9568K	ARMS	RMS_017P

ARMS, alveolar rhabdomyosarcoma; ERMS, embryonal rhabdomyosarcoma; NOS, not otherwise specified.

**Table 2:** Detected mutations in 60 rhabdomyosarcoma cases by targeted deep sequencing.

		**Histology**					**Fusion status**		**Methylation cluster**				
	**All (%)*****n*****=60**	**ERMS (%)*****n*****=35**	**ARMS (%)*****n*****=22**	**Mixed (%)*****n*****=1**	**NOS (%)*****n*****=1**	**U (%)*****n*****=1**	**FP (%)*****n*****=19**	**FN (%)*****n*****=41**	**E1 (%)*****n*****=11**	**E2 (%)*****n*****=11**	**A1 (%)*****n*****=18**	**A2 (%)*****n*****=10**	**NA (%)*****n*****=10**
*FGFR4/RAS/AKT* pathway total	24* (40)	16 (46)	7 (32)	1 (100)	0 (0)	0 (0)	3 (16)	21 (51)	6 (55)	9 (82)	3 (17)	2 (20)	4 (40)
*FGFR4*	5 (8.3)	5 (14)	0 (0)	0 (0)	0 (0)	0 (0)	0 (0)	5 (12)	0 (0)	4 (36)	0 (0)	0 (0)	1 (10)
* PTPN11*	2 (3.3)	2 (5.7)	0 (0)	0 (0)	0 (0)	0 (0)	0 (0)	2 (4.9)	0 (0)	2 (18)	0 (0)	0 (0)	0 (0)
* GAB1*	3 (5.0)	1 (2.9)	2 (9.1)	0 (0)	0 (0)	0 (0)	1 (5.3)	2 (4.9)	0 (0)	0 (0)	2 (11)	0 (0)	1 (10)
*PIK3CA*	3 (5.0)	2 (5.7)	1 (4.5)	0 (0)	0 (0)	0 (0)	1 (5.3)	2 (4.9)	0 (0)	2 (18)	0 (0)	0 (0)	1 (10)
*PTEN*	2 (3.3)	1 (2.9)	1 (4.5)	0 (0)	0 (0)	0 (0)	0 (0)	2 (4.9)	0 (0)	1 (9.1)	1 (5.6)	0 (0)	0 (0)
*HRAS*	1 (1.7)	0 (0)	1 (4.5)	0 (0)	0 (0)	0 (0)	0 (0)	1 (2.4)	0 (0)	0 (0)	0 (0)	1 (10)	0 (0)
*KRAS*	2 (3.3)	2 (5.7)	0 (0)	0 (0)	0 (0)	0 (0)	0 (0)	2 (4.9)	2 (18)	0 (0)	0 (0)	0 (0)	0 (0)
*NRAS*	7 (12)	5 (14)	2 (9.1)	0 (0)	0 (0)	0 (0)	1 (5.3)	6 (15)	2 (18)	2 (18)	1 (5.6)	0 (0)	2 (20)
*NF1*	3 (5.0)	2 (5.7)	0 (0)	1 (100)	0 (0)	0 (0)	0 (0)	3 (7.3)	1 (9.1)	2 (18)	0 (0)	0 (0)	0 (0)
													
*TP53*	9 (15)	6 (17)	1 (4.5)	0 (0)	1 (100)	1 (100)	1 (5.3)	8 (20)	0 (0)	5 (45)	2 (11)	1 (10)	1 (10)
*FBXW7*	1 (1.7)	1 (2.9)	0 (0)	0 (0)	0 (0)	0 (0)	0 (0)	1 (2.4)	0 (0)	1 (9.1)	0 (0)	0 (0)	0 (0)
*BCOR*	5 (8.3)	4 (11)	0 (0)	1 (100)	0 (0)	0 (0)	1 (5.3)	4 (9.8)	3 (27)	1 (9.1)	1 (5.6)	0 (0)	1 (10)
*ARID1A*	6 (10)	4 (11)	2 (9.1)	0 (0)	0 (0)	0 (0)	1 (5.3)	5 (12)	0 (0)	3 (27)	1 (5.6)	0 (0)	2 (20)
*ROBO1*	2 (3.3)	0 (0)	2 (9.1)	0 (0)	0 (0)	0 (0)	2 (11)	0 (0)	0 (0)	0 (0)	1 (5.6)	0 (0)	1 (10)

ARMS, alveolar rhabdomyosarcoma; ERMS, embryonal rhabdomyosarcoma; NOS, RMS not otherwise specified; U, unknown; FP, fusion positive; FN, fusion negative; NA, not available.

^*^4 cases had 2 mutations in *FGFR4/RAS/AKT* pathway genes.
