# Supplementary material for: Integrated genetic and epigenetic analysis defines novel molecular subgroups in rhabdomyosarcoma
Source: Nat Commun. 2015 Jul 3;6:7557. doi: 10.1038/ncomms8557 (PMC4506514; doi:10.1038/ncomms8557)
Supplement: Supplementary Figures — 1-10 [file ncomms8557-s1.pdf]

Supplementary Figure 1

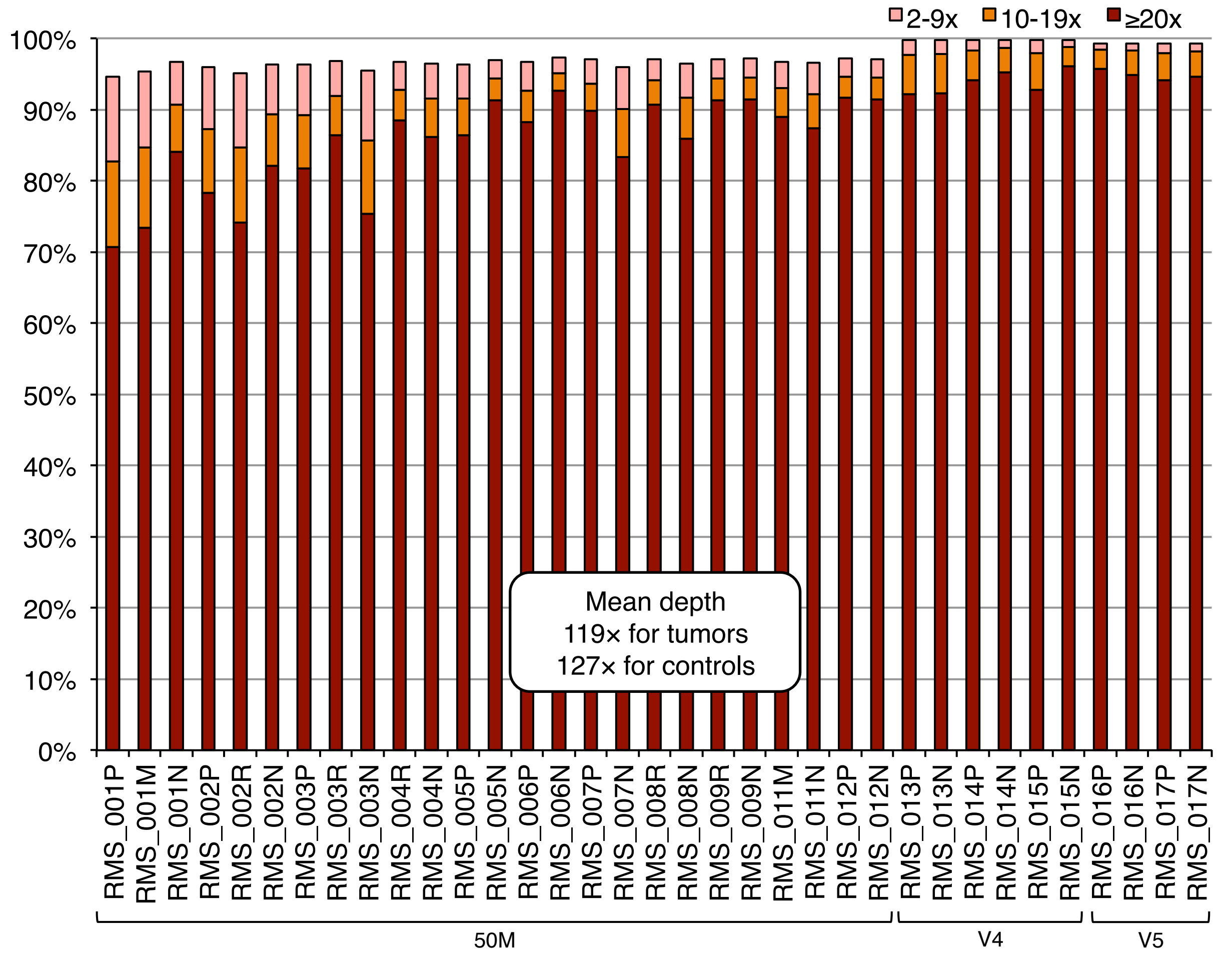

Mean coverage of whole exome sequencing in 16 RMS cases.

The coverage of the target regions analyzed at the indicated depth is plotted for each pair or trio. The mean depth for all tumor and normal samples is also indicated. Used bait is indicated blow sample ID.

P, primary; R, relapse; M, metastasis; N, normal.

Supplementary Figure 2

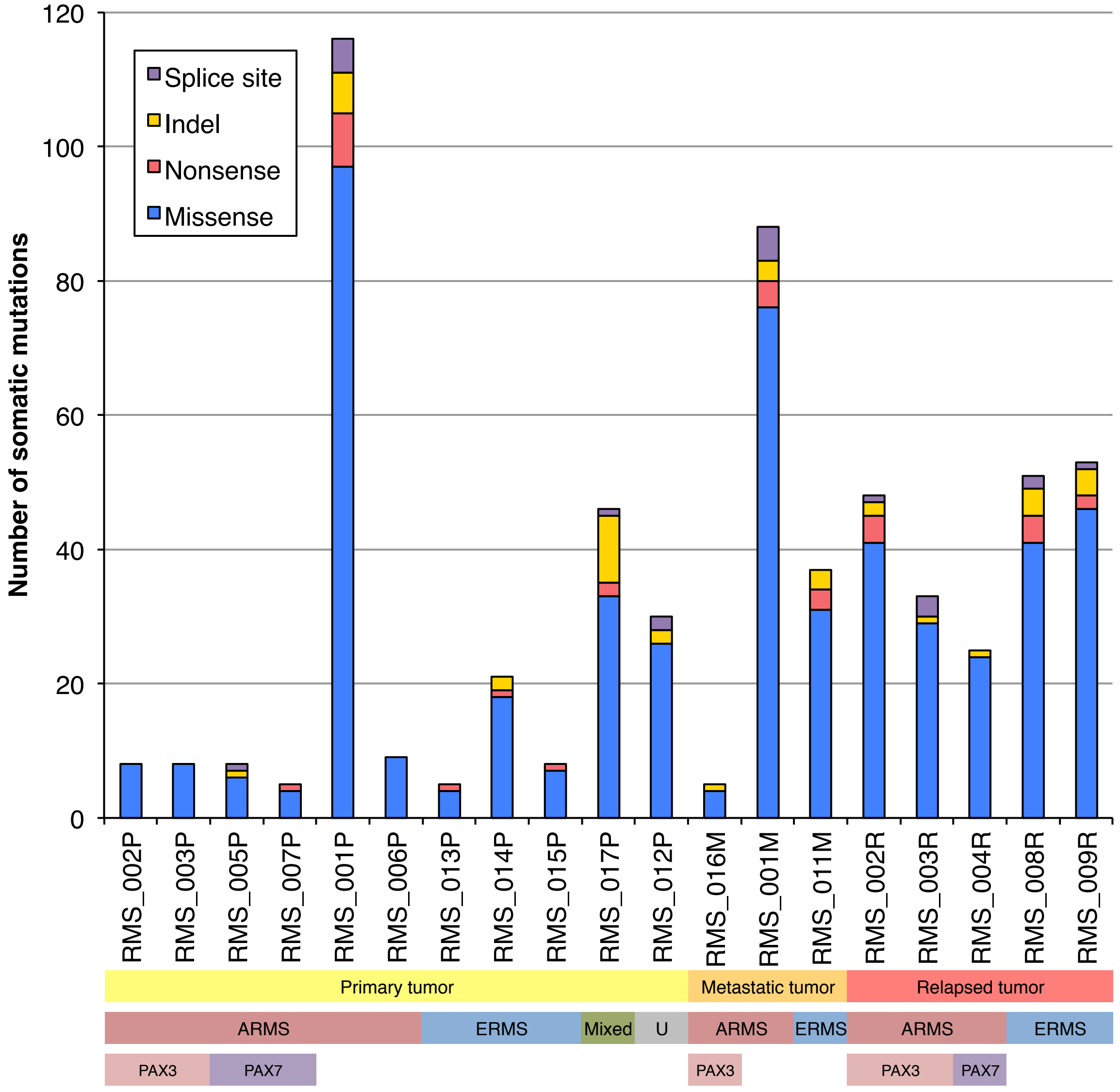

The number of validated somatic mutations detected by whole exome sequencing.

The number of validated somatic mutations and indels are displayed for each samples.
P, primary; R, relapse; M, metastasis; N, normal; M, mixed; U, unknown; PAX3, *PAX3-FOXO1* fusion positive; PAX7, *PAX7-FOXO1* fusion positive.

Supplementary Figure 3

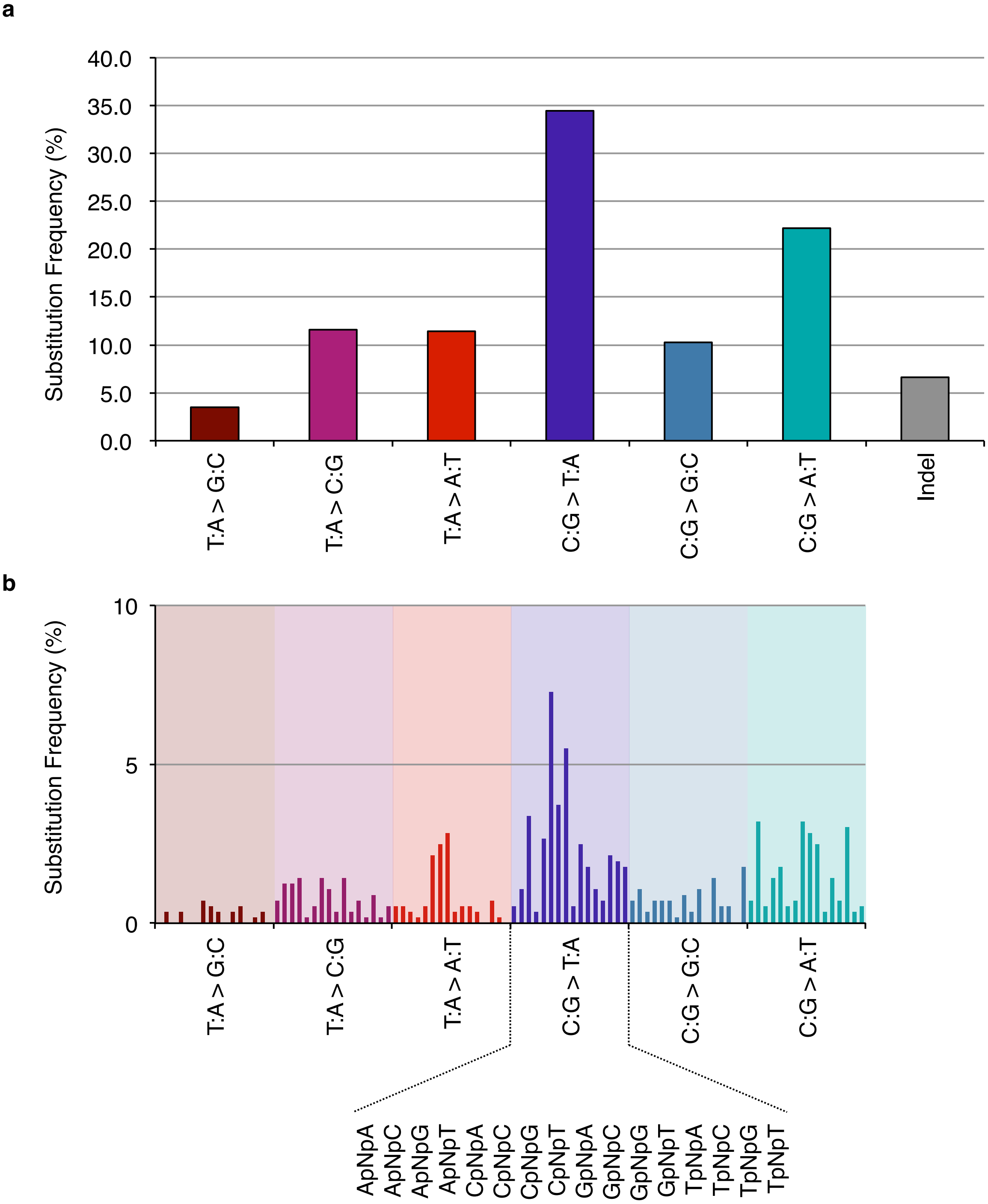

Distribution of substitutions in 16 different classes of validated mutations revealed by whole exome sequencing.

(a) Percentage of single nucleotide substitutions in 16 RMS cases.

(b) Trinucleotide contexts in 16 RMS cases. Each signature is displayed according to the 96 substitution classification. The six types of substitutions are displayed in different colors. Each color bar shows 16 types signatures with immediately 3’ and 5’ side nucleotide to the mutated base “N”.

Supplementary Figure 4

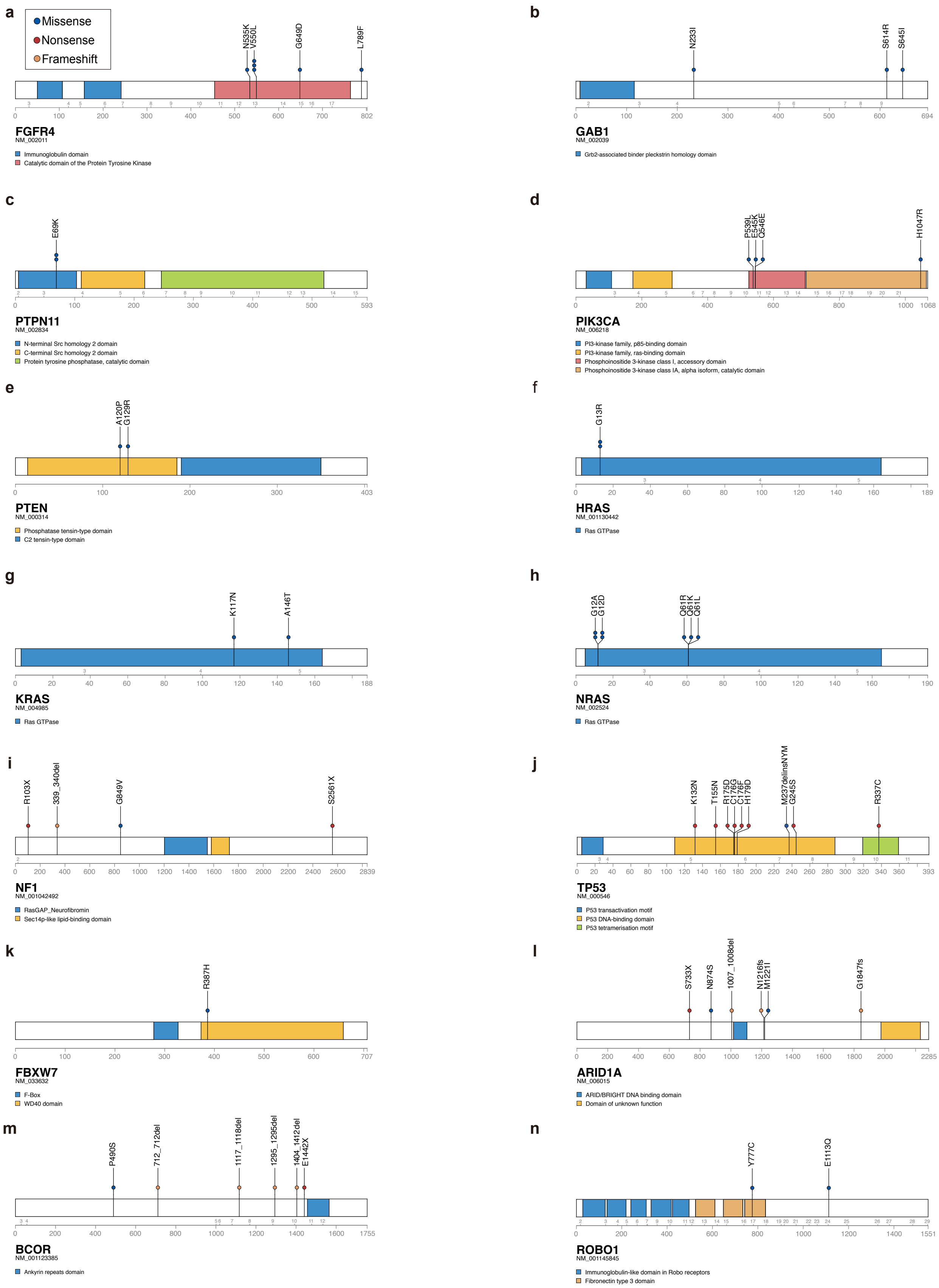

Distribution of mutated genes in 60 RMS cases by targeted deep amplicon sequencing.

Types of mutations are distinguished by the indicated colors. These figures were created using Protein painter (http://explorepcgp.org/proteinPainter).

Supplementary Figure 5

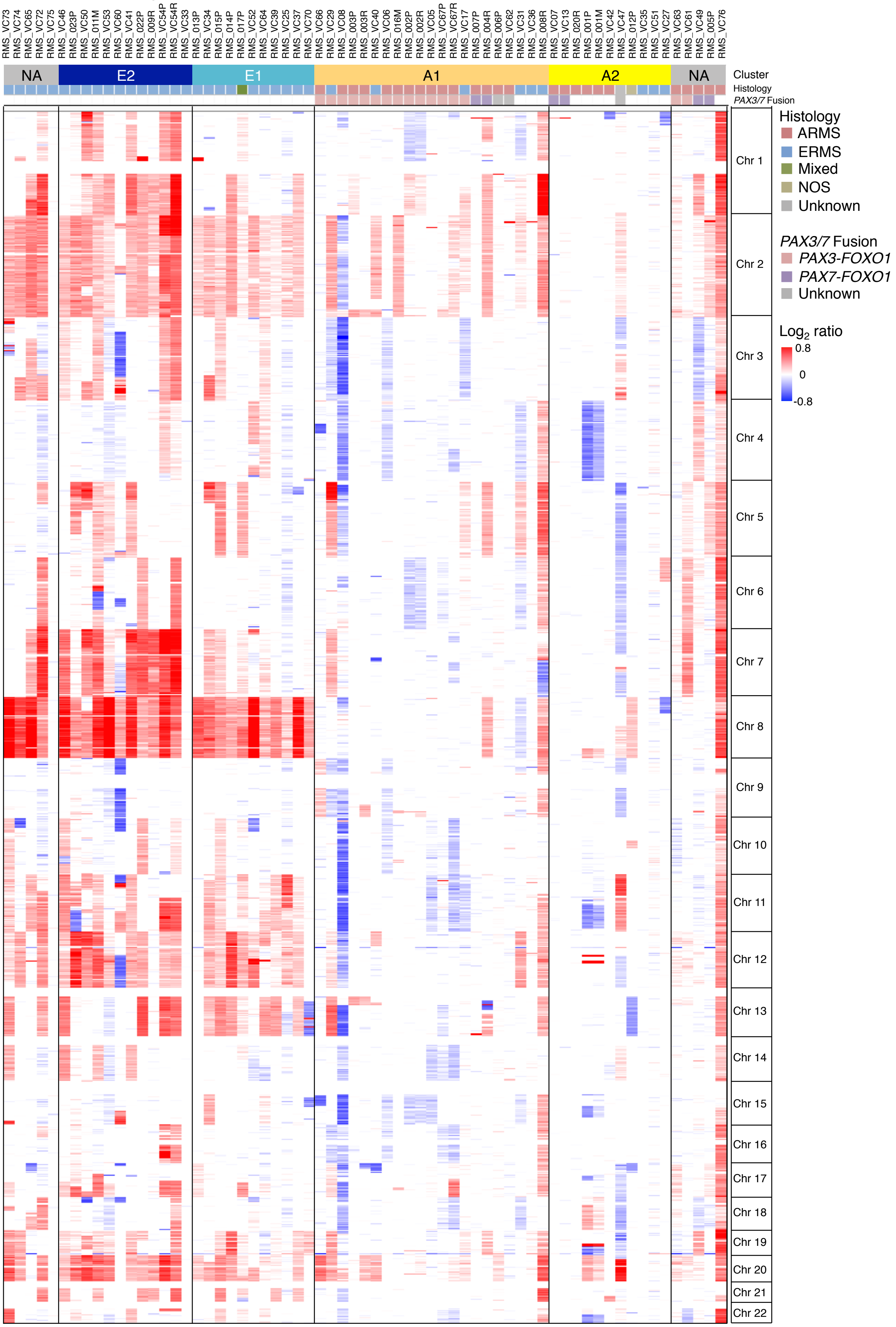

Log-ratio copy number heat map in 65 RMS samples.

Copy number data obtained from 250K or Cytoscan array is displayed by a color gradient based on CNAG output for 65 RMS samples. NA, Methylation data not available

Supplementary Figure 6

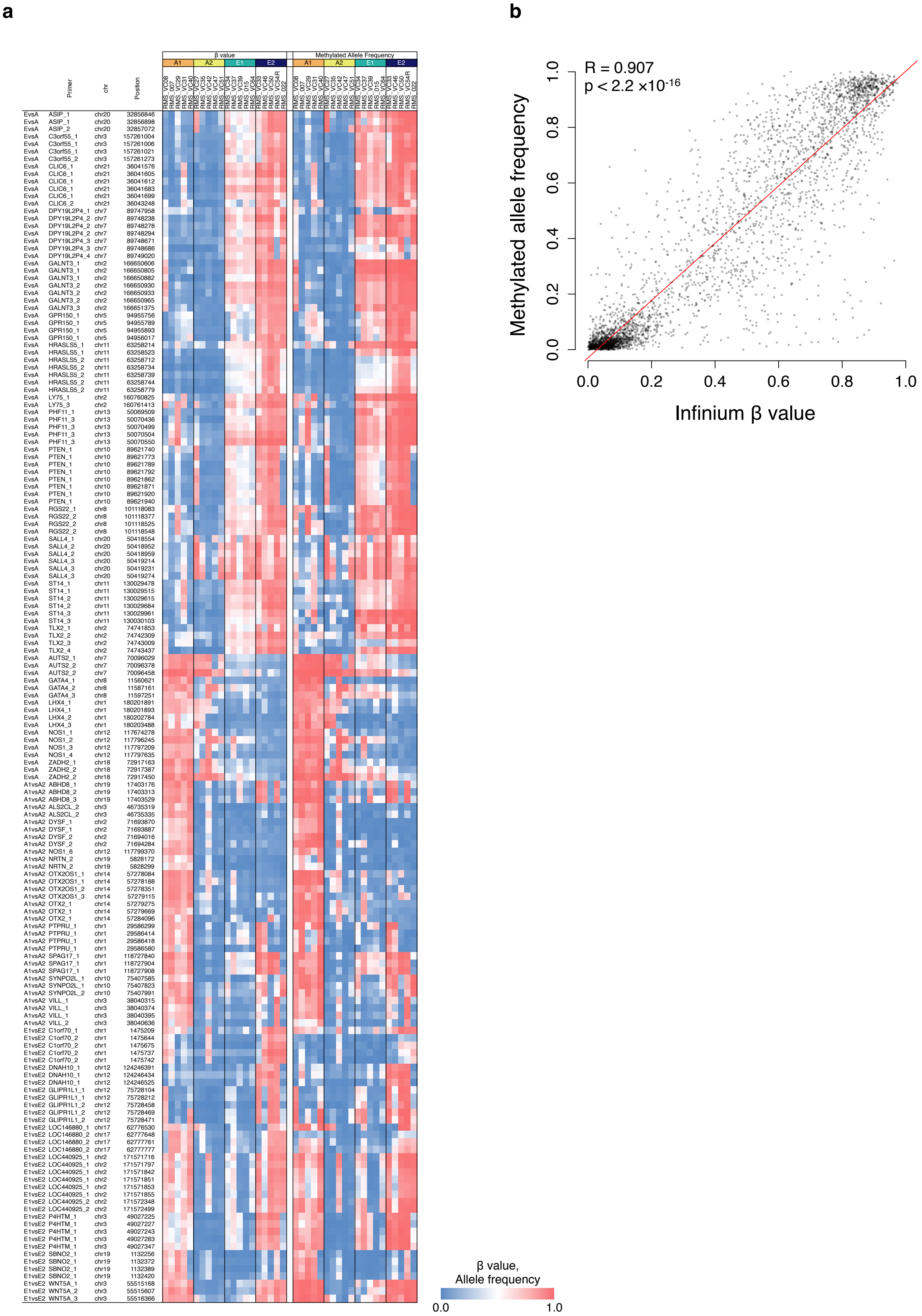

Validation of detected methylation beta values.

(a) Heatmap of beta values and methylated allele frequencies in validated probes.
Beta values heat map of DNA methylation array is shown on left side, and methylated allele frequencies heat map of bisulfite targeted deep sequencing is shown on right side.

(b) Scatter plot of beta value and methylated allele frequency.
The red line indicates a linear regression. Infinium beta values and methylated allele frequencies detected by bisulfite deep sequence were significantly correlated (The spearman correlation coefficient = 0.902, p value < 2.2×10^{-16}).

Supplementary Figure 7

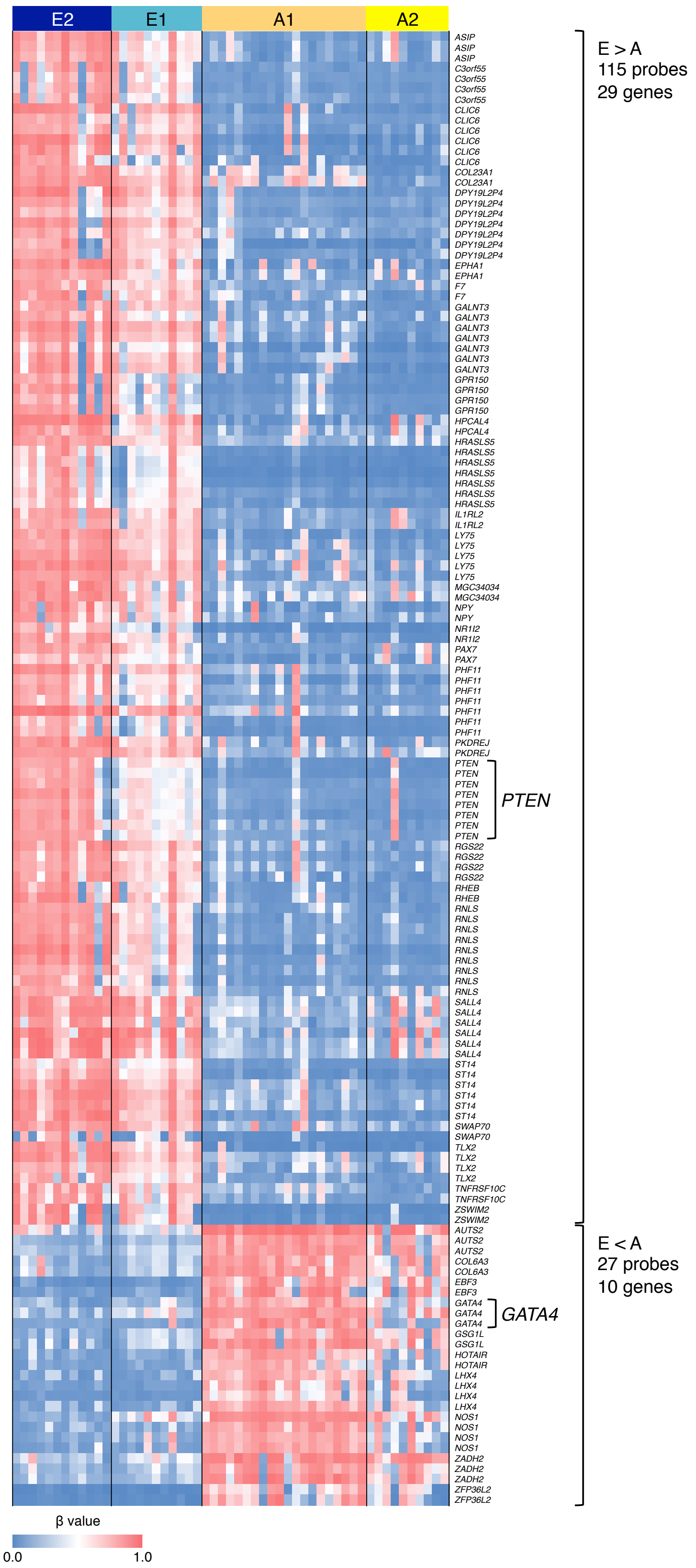

A heat map of differentially methylated probes between cluster A1/A2 and E1/E2.

Methylation status of 142 differently methylated genes between the two clusters (Wilcoxon rank sum test p values $<10^{-8}$) is displayed. Genes with only 1 probe are excluded. Probes and genes are listed in Supplementary Table 8.

Supplementary Figure 8

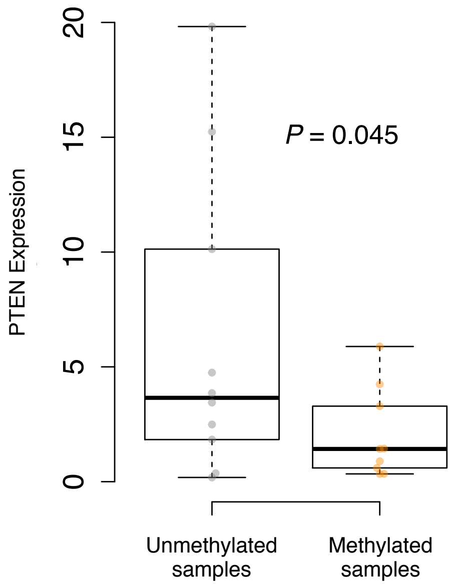

Correlation between methylation in promoter region and expression of PTEN.

Expression analysis of PTEN by quantitative PCR in methylated (n = 9) and unmethylated (n = 10) samples. Methylated samples show lower expression (*t*-test P value = 0.0045). For the box and whisker plots, the bottom and top of the box are the first and third quartiles, the line inside the box is the median, and the whiskers extend up to 1.5 times the interquartile range.

Supplementary Figure 9

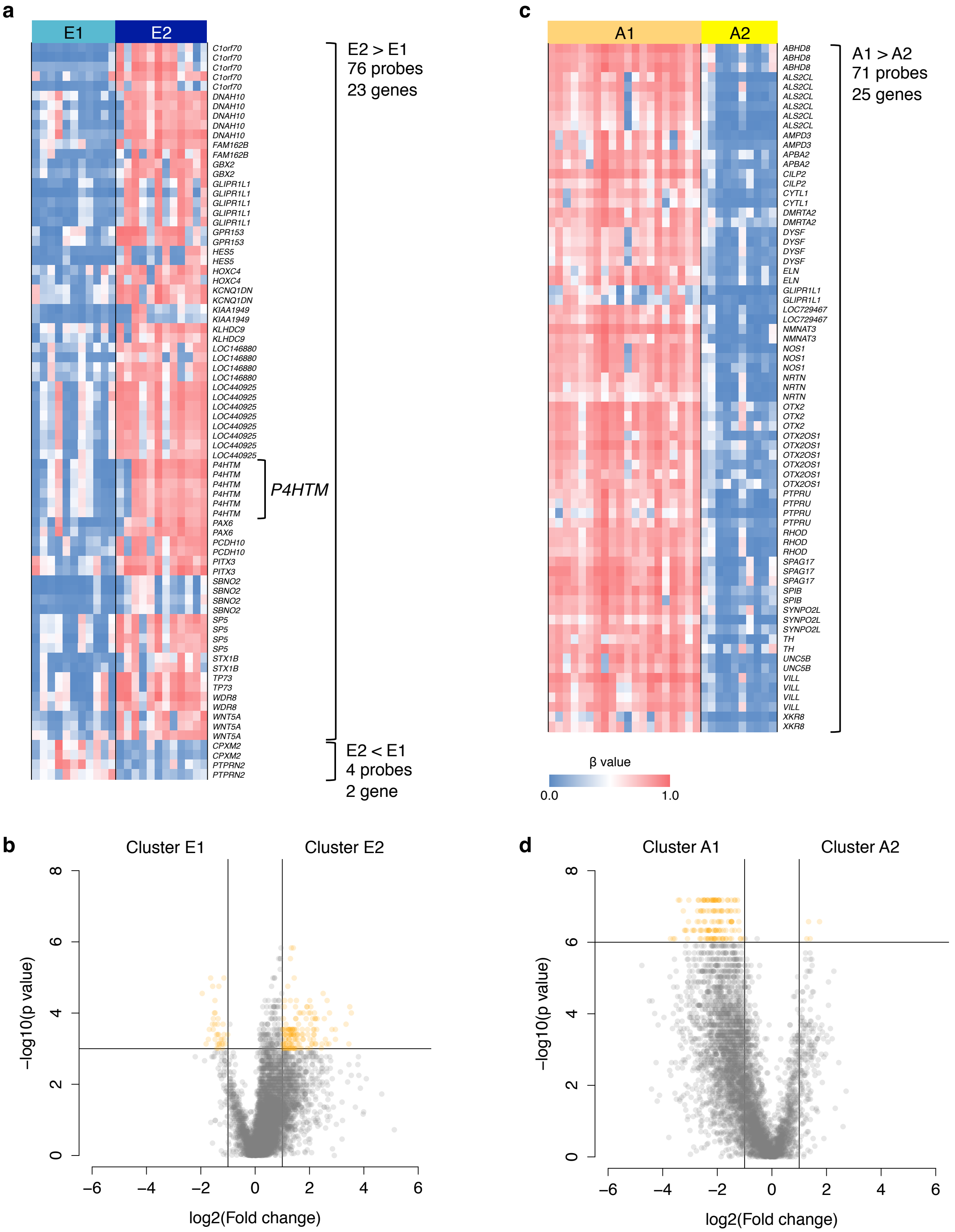

Differentially methylated probes between E1 vs. E2 and A1 vs. A2.

(a, b) Methylation status of 76 differently methylated genes between E1 and E2 (p value $<10^{-3}$) and A1 and A2 (p value $<10^{-6}$) is displayed. Genes with only 1 probe are excluded. (c, d) Volcano plot analysis (-log10-transformed P value vs. log2-fold change) is shown. The P values were calculated using the Wilcoxon rank sum test. Probes and genes are listed in Supplementary Table 9 and 10.

Supplementary Figure 10

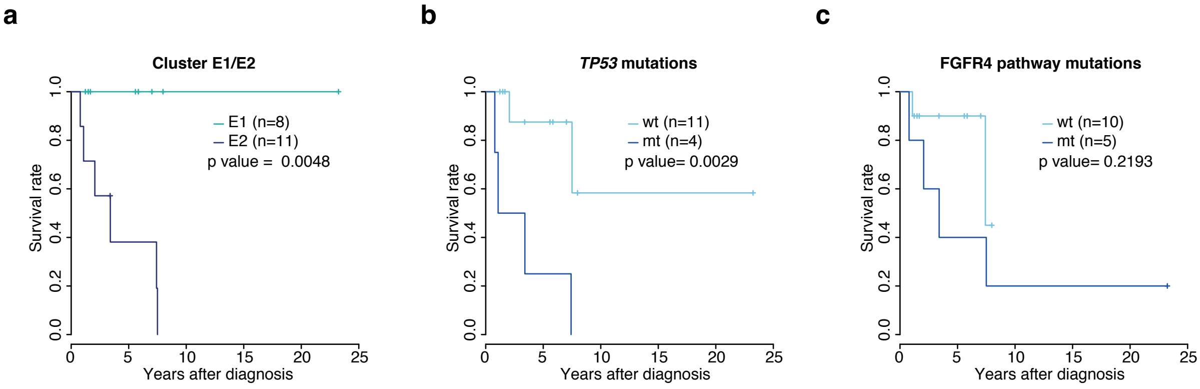

Comparison of outcome in cluster E with methylation cluster or genetic alterations

Kaplan-Meyer survival curves in cluster E samples according to methylation cluster, *TP53* status, and FGFR4 pathway status. (a) Methylation cluster (E1 vs. E2), (b) *TP53* status (*TP53* wild type vs. *TP53* mutation), and (c) FGFR4 pathway status (FGFR4 pathway wild type vs. FGFR4 pathway mutation). The P values were calculated by log-rank test. FGFR4 pathway mutations are including mutations in *FGFR4, PTPN11, PIK3CA, PTEN*. wt, wild type; mt, mutation.
